# Laser Wire Directed Energy Deposition of 5356 Aluminum Alloy: Process Parameter Optimization and Porosity Prediction

**DOI:** 10.3390/ma19061104

**Published:** 2026-03-12

**Authors:** Xiangfei Zhang, Yujia Mei, Huomu Yang, Shouhuan Zhou

**Affiliations:** College of Electronics and Information Engineering, Sichuan University, Chengdu 610065, China; 18202883187@163.com (X.Z.); 17802379558@163.com (Y.M.); zhoush@scu.edu.cn (S.Z.)

**Keywords:** laser wire directional energy deposition, 5356 aluminum alloy, porosity, machine learning

## Abstract

Laser wire directed energy deposition (LWDED) has garnered significant attention for the fabrication of large metallic components. However, the complex coupling effects among its process parameters pose challenges for porosity control. Optimizing parameter combinations to effectively minimize porosity is therefore critical to the broader adoption of this technology. In this study, systematic experiments and modeling were conducted to optimize the LWDED process parameters and predict porosity. First, single-factor and orthogonal experiments were performed to evaluate the individual effects of laser power, scanning speed, wire feeding speed, and air pressure on porosity. Subsequently, range analysis and analysis of variance were employed to determine the influence of each parameter and the significance of their interactions. Four machine learning models—SVR, RF, GPR, and XGBoost—were then trained and compared. Among them, the SVR model exhibited the best predictive performance, achieving an R^2^ of 0.8960, an RMSE of 0.19, and an MAE of 0.15, outperforming the other three models. Based on this, the SVR model was further utilized to establish the mapping between process parameters and porosity. Contour maps and three-dimensional surface plots were generated to visualize porosity variation patterns under interacting parameters. Validation experiments showed that the maximum relative error between model predictions and experimental measurements was 0.514%, with an average error of 0.251%. This study provides a reliable reference for selecting low-porosity parameter combinations in the LWDED fabrication of 5356 aluminum alloy components.

## 1. Introduction

Currently, industries such as aerospace, automotive manufacturing, and rail transit are undergoing rapid technological advancement, driving an increasing demand for large metallic components that offer both lightweight and high-strength properties. 5356 aluminum alloy, a representative aluminum-magnesium alloy, is characterized by low density, high strength, and excellent corrosion resistance [1], making it an ideal candidate for the fabrication of large, lightweight, and geometrically complex components [2]. Laser wire directed energy deposition (LWDED), a key branch of additive manufacturing (AM), offers distinct advantages, including high material utilization, superior deposition efficiency, and the capacity to produce large-scale structures [3]. These attributes render LWDED highly promising for the efficient fabrication of large 5356 aluminum alloy components. Consequently, the application of LWDED in the production of high-performance large aluminum alloy parts has emerged as a research focus within the field of additive manufacturing.

LWDED technology for fabricating 5356 aluminum alloy components is prone to producing metallurgical pores and process-induced pores [4]. Pore defects can significantly reduce key properties such as hardness, shear strength, and corrosion resistance of components. In severe cases, they can lead to component failure [5]. The solubility of hydrogen in liquid aluminum increases with temperature. When the molten pool enters the solidification stage, where temperature drops rapidly, hydrogen solubility decreases. Hydrogen is then released from liquid aluminum to form bubbles [6]. Bubbles that fail to escape in time are trapped and form metallurgical pores. The formation of process-induced pores is closely related to the stability of the keyhole during laser deep penetration welding. When the keyhole tip is sealed by molten metal flow, it separates to form process-induced pores [7]. Research shows that pores can be reduced by optimizing process parameters, but the mechanism by which the LWDED process parameters control pore formation is complex [8]. For example, laser power reduces porosity by maintaining keyhole stability [9]. Scanning speed reduces porosity by controlling the molten pool solidification rate [10]. Wire feeding speed reduces porosity by controlling the wire transfer mode to maintain molten pool and keyhole stability [7,11]. Air pressure controls shielding gas flow to prevent impurities from ambient air from being drawn into the molten pool, thereby reducing porosity [12]. Additionally, interactions exist among these parameters. For instance, the interaction between laser power and scanning speed affects the balance between heat input and solidification rate. The interaction between laser power and wire feeding speed changes the energy-to-material ratio. These interactions significantly increase the difficulty of optimizing parameter combinations to reduce porosity. Therefore, effectively optimizing process parameters to fabricate low-porosity aluminum alloy components is a current research focus.

Establishing a mapping relationship between process parameter combinations and porosity is key to achieving high-quality printing of 5356 aluminum alloy via LWDED. Traditional process optimization relies primarily on statistical modeling methods, such as analysis of variance (ANOVA), response surface methodology (RSM), and the Taguchi method, which build predictive models between process parameters and target outputs from limited experimental data. Javidrad et al. [13] combined ANOVA and RSM for multi-objective optimization, successfully identifying optimal laser power, scanning speed, and powder feed rate for laser powder directed energy deposition (LPDED) of Inconel 738LC, balancing porosity against deposition efficiency. Xiao et al. [14] applied Taguchi experimental design to co-optimize laser power, scanning speed, powder feed rate, and shielding gas flow rate, improving the density of B4C/Al composites fabricated by LPDED. In fused deposition modeling (FDM), Saad et al. [15] combined RSM with particle swarm optimization (PSO) to optimize process parameters for flexural strength. Although these traditional methods are technically mature, they still depend on accumulated experience and extensive trial-and-error experiments [16], and struggle to handle the complexity of multi-parameter coupling [17].

In recent years, machine learning (ML) has shown great potential in process optimization and defect prediction for additive manufacturing, owing to its strong nonlinear fitting capability and ability to extract complex features [18,19]. Existing studies have covered a wide range of processes, including wire arc additive manufacturing (WAAM), fused deposition modeling (FDM), laser powder directed energy deposition (LPDED), and laser powder bed fusion (LPBF). Researchers have used algorithms such as GPR, XGBoost, NN, ANN, and GBDT to build models linking process parameters to part geometry [20], surface quality [21], relative density [22,23,24], flexural strength [15], and porosity [25,26]. Furthermore, contour plots and 3D surface visualizations have enabled researchers to intuitively reveal the interactions among multiple parameters. Combined with heuristic search methods such as PSO, self-adaptive harmony search (SAHS), and genetic algorithms (GA), these approaches have enabled precise identification of optimal parameter combinations within constrained search spaces.

For LWDED specifically, Liu et al. [27] used GPR and a naive Bayes classifier to systematically predict the geometric features, microstructure, and surface quality of Ti-6Al-4V deposited layers. Abuabiah et al. [28] established a regression model via ANOVA to optimize the deposition quality of Inconel 718. However, despite the widespread application of these optimization frameworks across various additive manufacturing processes, no study has yet reported machine learning-based porosity modeling or contour plot analysis for 5356 aluminum alloy fabricated by LWDED. Given the considerable application potential of LWDED technology and 5356 aluminum alloy, it is both necessary and timely to draw on established optimization frameworks from the broader additive manufacturing field and introduce machine learning for porosity-oriented process optimization in LWDED.

This study takes the development of low-porosity 5356 aluminum alloy LWDED components as the core objective and conducts process parameter optimization research. First, the variation patterns of porosity under the independent action of core parameters such as laser power, scanning speed, wire feeding speed, and air pressure are clarified through the controlled variable method. Then, the optimal process parameter combination is screened through orthogonal experiments. Range analysis and variance analysis are combined to reveal multi-parameter interaction mechanisms and clarify the dominant role of laser power and the significance of key interaction terms. Finally, a machine learning prediction model is built based on experimental data. Contour maps and 3D surface plots are used to show the variation patterns of porosity under multi-parameter interactions.

## 2. Experimental Methods

As mentioned above, the four process parameters of laser power, scanning speed, wire feeding speed, and air pressure are particularly important for their effects on pores. Besides these core parameters, process parameters such as wire feed angle and defocusing distance also affect forming quality. However, these parameters are more oriented toward “forming accuracy” rather than being dominant factors for “pore defects”. Their optimization ranges are also narrow. For example, wire feed angle selection is generally 15°, 30°, 45°, 60°, and 75°. Existing research has confirmed that 45° is the optimal wire feed angle [3]. Therefore, the wire feed angle is fixed at 45°. Defocusing distance can be divided into three cases: positive defocusing, negative defocusing, and zero defocusing [29]. Positive defocusing provides better forming quality with uniform and attractive morphology. Therefore, positive defocusing is selected with a focal distance of 5 mm. In summary, the four core process parameters of laser power, wire feeding speed, scanning speed, and air pressure are selected for experiments to study their influence patterns on the porosity of 5356 aluminum alloy LWDED. The experimental setup and porosity detection method will be described in detail below.

### 2.1. Experimental Setup

**Figure 1:**
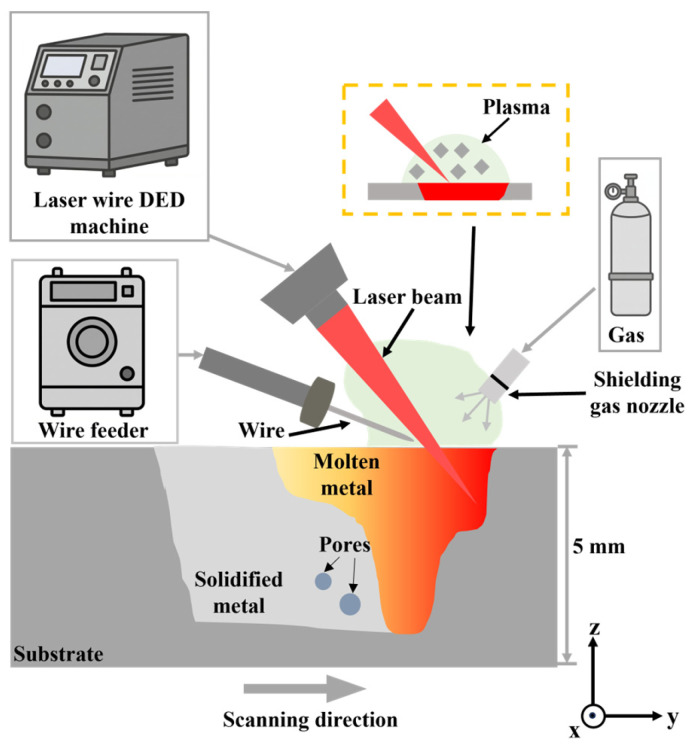
The LWDED experimental setup.

The LWDED equipment (Han’s Laser, Shenzhen, China) used in the experiment is shown in Figure 1. It mainly consists of the LWDED system, wire feeder, and shielding gas system. The LWDED system includes a fiber laser (output wavelength 1070 nm) and a three-dimensional moving platform. The metal wire material selected is 5356 aluminum alloy wire with a diameter of 0.8 mm. The substrate is a 5356 aluminum alloy plate with dimensions of 100 mm × 50 mm × 5 mm. The chemical element composition of both is shown in Table 1 [30]. Before the experiment, anhydrous ethanol was used to clean oil and moisture from the substrate surface to avoid impurities being drawn into the molten pool during deposition to form pores. The wire is fed into the molten pool through off-axis wire feeding. To avoid damage to the laser head from aluminum alloy reflecting the laser, the laser beam is focused on the substrate surface at a 45° inclination angle to form a molten pool. Throughout the deposition process, high-purity argon gas is used as shielding gas. It can both prevent component oxidation and blow away plasma that accumulates above the molten pool during deposition.

### 2.2. Porosity Detection Method

**Figure 2:**
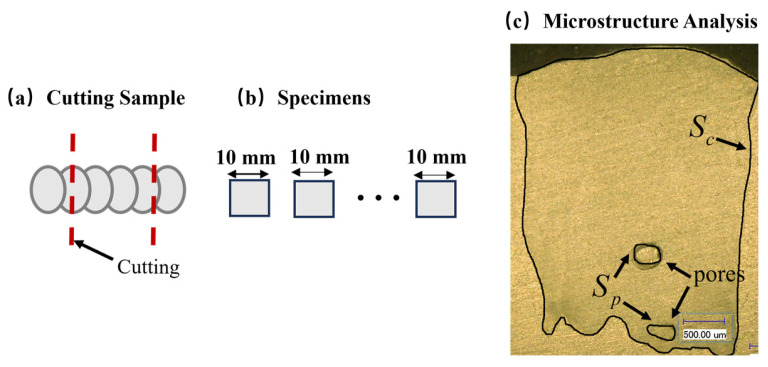
Porosity detection method. (**a**,**b**) Schematic diagram of the sample cutting method; (**c**) Cross-section of the sample.

Pore defects seriously affect the mechanical properties and service reliability of components. Therefore, porosity detection and evaluation are core aspects of quality assessment for additive manufacturing components. Porosity detection methods are mainly divided into two categories: non-destructive testing and destructive testing. Non-destructive testing includes X-ray computed tomography (XCT) and ultrasonic testing [31]. Destructive testing mainly uses metallographic sectioning combined with microscopic observation [32]. Compared to the high cost and time consumption of non-destructive methods, this experiment uses the low-cost metallographic sectioning method to detect porosity. To ensure the reliability of measurement results, the porosity of multiple cross-sections is measured, and the average value is taken as the final porosity of the sample. The specific process is shown in Figure 2. The sample prepared with each set of process parameters is cut along the cross-section into 5 segments, each about 10 mm, as shown in Figure 2a,b. After hot mounting, they are ground sequentially with 400-grit, 800-grit, and 1500-grit sandpaper, then polished with 50 nm silica suspension. The polished surface is then etched with Keller’s reagent for 30 s. The cross-section of the sample is observed through an optical microscope, as shown in Figure 2c. Finally, ImageJ (Version 1.54g, National Institutes of Health, Bethesda, MD, USA) image analysis software is used to obtain the cross-sectional area and pore area of the sample [33]. The porosity calculation formula refers to the report by Li et al. [32], shown in Equation (1):

(1) $$Porosity=\frac{S_{p}}{S_{c}}\times 100\;\%$$

where $s_{p}$ represents the total area of the pores and $s_{c}$ denotes the total cross-sectional area.

## 3. Influence of Process Parameters on Porosity

Before conducting experimental investigations into the effects of process parameters on porosity, it is necessary to establish a physical metallurgy foundation for understanding the fundamental mechanisms of pore formation during the LWDED process. For the 5356 Al-Mg alloy, porosity formation is not governed by a single factor but results from the multi-field coupling of melt pool dynamics, keyhole stability, gas entrapment, and solidification behavior.

Laser power provides the thermal energy required to melt the substrate surface and establish a molten pool into which the fed wire is deposited. Laser power, together with scanning speed, determines the energy input. Due to the high reflectivity and high thermal conductivity of aluminum alloys, a sufficiently high laser power is required to maintain a stable molten pool. However, the 5356 alloy contains approximately 5% magnesium (Mg). Owing to the low boiling point of Mg, excessive laser power readily triggers intense vaporization of Mg elements. This vaporization not only causes loss of alloying elements but also generates strong metal vapor recoil pressure, which severely destabilizes the keyhole. Violent fluctuations or intermittent collapse of the keyhole can cause the keyhole tip to be closed off by molten metal flow, thereby forming pores. Simultaneously, scanning speed governs the solidification dynamics and bubble escape kinetics. Hydrogen exhibits high solubility in high-temperature liquid aluminum. When the scanning speed is excessively high, the solidification rate increases dramatically, and the solidification front advances rapidly. Consequently, precipitated hydrogen bubbles and entrapped shielding gas are “frozen” in the deposited layer before they can float out, leading to gas entrapment. Conversely, appropriately reducing the scanning speed significantly shortens the solidification time of the melt pool, providing sufficient time for bubbles to float and escape.

Wire feeding speed not only determines the deposition rate but also controls the molten metal transfer mode. If the wire feeding speed is mismatched with the available heat input (e.g., high feed rate with insufficient energy), it can cause the wire to directly strike the melt pool or result in large droplets detaching into the pool. These unstable transfer modes induce severe mechanical disturbances on the melt pool surface, mechanically entraining external shielding gas or even ambient air into the interior. Additionally, an excessively high wire feeding speed introduces more hydrogen (from moisture or oxides absorbed on the wire surface) into the melt pool per unit time, directly increasing the probability of metallurgical pore formation.

Air pressure directly affects the gas flow conditions above the melt pool. When the air pressure is insufficient to effectively shield the molten pool from the surrounding atmosphere, it leads to oxidation of the melt pool surface and entrainment of hydrogen from the air, triggering extensive hydrogen porosity. However, when the air pressure is too high, the high-velocity gas jet impinges on the melt pool surface, disrupting its steady state and entraining shielding gas into the molten pool, thereby forming pores.

To clarify the independent influence patterns of laser power, scanning speed, wire feeding speed, and air pressure on the porosity of 5356 aluminum alloy samples prepared by LWDED, as well as the influence weight of each parameter and the significance of interaction terms, single-factor experiments and orthogonal experiments were conducted.

### 3.1. Single-Factor Experiments

The independent effects of individual process parameters on porosity are studied by varying the target parameter while maintaining the remaining three at constant levels. These single-factor experiments would establish the empirical foundation for the subsequent orthogonal experimental design and the development of predictive models. The ranges and increments of the parameters were defined as follows: laser power ranged from 500 W to 1700 W in increments of 100 W. Wire feeding speed ranged from 80 cm/min to 200 cm/min in increments of 10 cm/min, scanning speed was adjusted from 400 mm/min to 1000 mm/min in 50 mm/min increments, and Air pressure was varied between 0.05 MPa and 0.4 MPa at designated intervals.

#### 3.1.1. Influence of Laser Power

Laser power governs the stability of keyhole behavior and the thermal distribution within the melt pool, thereby serving as the primary determinant of porosity formation in the LWDED process [9]. In the analysis, scanning speed, wire feeding speed, and Air pressure were fixed at 850 mm/min, 130 cm/min, and 0.24 MPa, respectively. Preliminary trials indicated that laser power levels below 700 W provided insufficient energy to fully melt the wire, resulting in wire adhesion to the substrate. Conversely, levels exceeding 1700 W caused excessive penetration. Consequently, this work focuses on the influence of laser power ranging from 700 to 1700 W.

**Figure 3:**
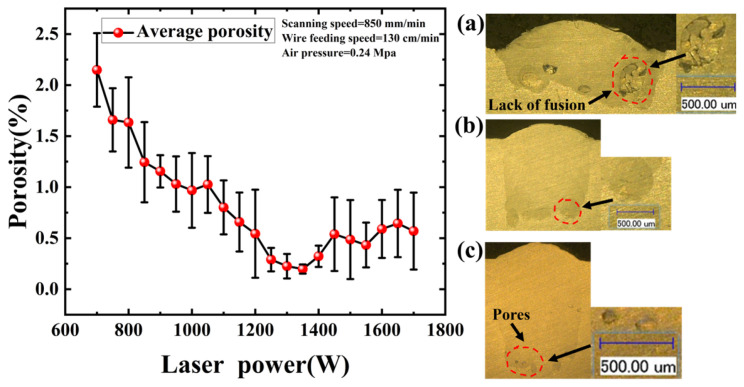
Influence of Laser Power on Porosity. (**a**) 850 W; (**b**) 1350 W; (**c**) 1600 W.

As shown in Figure 3, porosity exhibited at a relatively high level at 700 W. This is attributed to insufficient heat input failing to achieve adequate fusion between the wire and the substrate, resulting in substantial lack-of-fusion defects and a shallow penetration depth under this condition [34]. As laser power increased, molten metal progressively filled these void regions, and the melt depth deepened, resulting in a downward trend in porosity. The minimum porosity was achieved at 1350 W. In this regime, the wire metal transfer mode stabilized into a liquid bridge transfer mode characterized by a stable keyhole morphology, which allowed entrapped gas sufficient time to escape the melt pool [11]. However, as laser power exceeded 1350 W, a slight increase in porosity was observed. This phenomenon arises from excessive energy input, inducing keyhole oscillation. Intense vapor recoil pressure causes the periodic collapse of the keyhole walls, generating gas bubbles that are entrapped prior to solidification and manifest as pores [4].

#### 3.1.2. Influence of Scanning Speed

Scanning speed serves as a critical process parameter in LWDED by governing the laser-material interaction time. It determines the solidification rate of the molten pool, thereby influencing gas escape efficiency and porosity formation [10]. In this work, laser power, wire feeding speed, and Air pressure were fixed at 1350 W, 130 cm/min, and 0.24 MPa, respectively. Preliminary trials indicated that scanning speeds below 450 mm/min resulted in excessive heat input, causing the melt pool to penetrate through the substrate. Consequently, this study focuses on scanning speeds ranging from 450 mm/min to 1150 mm/min.

**Figure 4:**
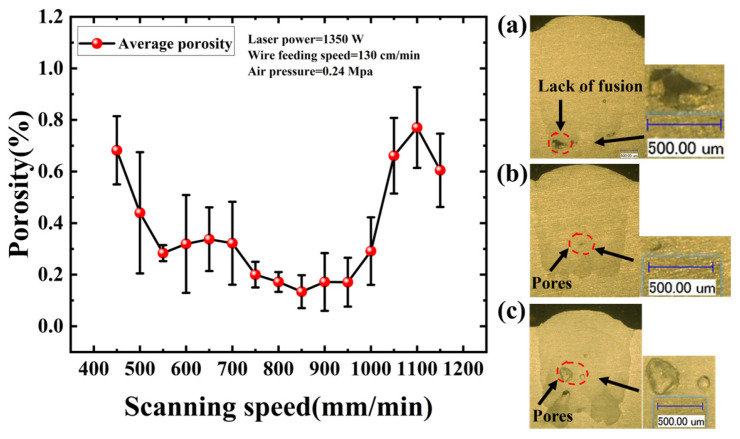
Influence of Scanning Speed on Porosity. (**a**) 500 mm/min; (**b**) 850 mm/min; (**c**) 1100 mm/min.

As depicted in Figure 4, when the scanning speed is set at 450 mm/min, the porosity is exhibited at a high level. This is attributed to the prolonged interaction between the laser and the substrate, which significantly increased the heat input per unit area. This excessive energy induced intense convective flow and fluctuations within the melt pool, exacerbating instability and facilitating the entrainment of ambient gas [35]. As scanning speed was progressively increased, the reduced laser interaction time brought the heat input to a reasonable level, thereby enhancing melt pool stability and leading to a continuous decline in porosity. The porosity reached a minimum at 850 mm/min; at this velocity, the solidification rate was moderate, affording gas bubbles sufficient time to float to the surface and escape prior to solidification. However, scanning speeds exceeding 850 mm/min resulted in a resurgence of porosity. This trend arises because excessive speeds shorten the solidification window [36], causing gas bubbles generated within the melt pool to become entrapped before they can escape. Furthermore, high scanning speeds may lead to insufficient interaction between the wire and the melt pool, increasing the incidence of localized lack-of-fusion defects and further elevating porosity.

#### 3.1.3. Influence of Wire Feeding Speed

Wire feeding speed is the primary determinant of the material deposition rate in the LWDED process. By dictating the volume of material input per unit time, it influences melt pool stability and wire metal transfer mode, which ultimately influence porosity formation [7,11]. In this work, laser power, scanning speed, and air pressure were fixed at 1350 W, 850 mm/min, and 0.24 MPa, respectively. Preliminary observations indicated that wire feeding speeds below 80 cm/min resulted in discontinuous deposition characterized by the balling phenomenon. Conversely, rates exceeding 220 cm/min caused the wire to enter the melt pool prior to complete fusion, preventing effective fabrication. Consequently, this study investigates the influence of wire feeding speeds ranging from 80 cm/min to 220 cm/min.

**Figure 5:**
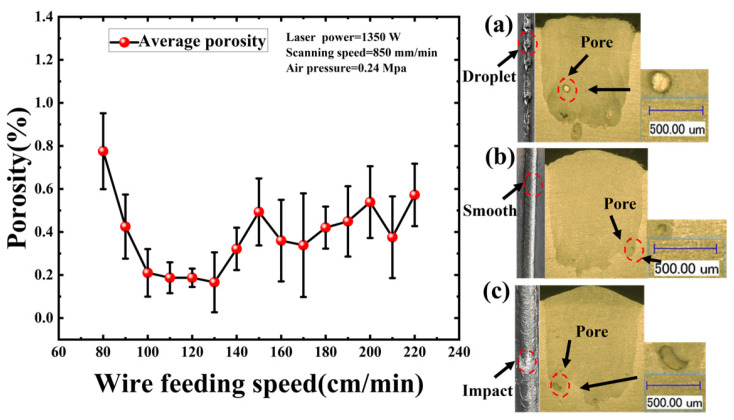
Influence of Wire Feeding Speed on Porosity. (**a**) 80 cm/min; (**b**) 130 cm/min; (**c**) 180 cm/min.

As illustrated in Figure 5, porosity exhibited at a relatively high level at 80 cm/min. In this condition, the wire melts prematurely before reaching the melt pool. Driven by the combined effects of surface tension and gravity, the molten wire tip contracts into spherical droplets that enter the pool via a dripping mechanism, characteristic of the droplet transfer mode [37]. The periodic impact of these droplets induces severe oscillation within the melt pool, promoting gas entrainment. Furthermore, this instability contributes to balling defects on the component surface; collectively, these factors result in increased porosity. As the wire feeding speed was increased, a stable liquid bridge formed between the molten wire tip and the melt pool surface, enabling continuous metal transfer via the liquid bridge transfer mode [38]. This transition stabilized melt pool dynamics and produced a smooth surface morphology, leading to a marked reduction in porosity. Porosity reached a minimum at 130 cm/min. However, feed rates exceeding 130 cm/min resulted in a renewed increase in porosity. This trend is attributed to excessive feed rates, causing the wire to impact the melt pool prior to complete melting. The unmelted wire exerts a mechanical impact on the melt pool, destabilizing the stability of the melt pool and inducing spatter, which consequently elevates porosity.

#### 3.1.4. Influence of Air Pressure

Air pressure regulates the shielding gas flux in the LWDED process. It prevents the oxidation of the melt pool and the entrainment of impurities into it, thereby governing porosity formation [12]. In this work, laser power, scanning speed, and wire feeding speed were fixed at 1350 W, 850 mm/min, and 130 cm/min, respectively.

**Figure 6:**
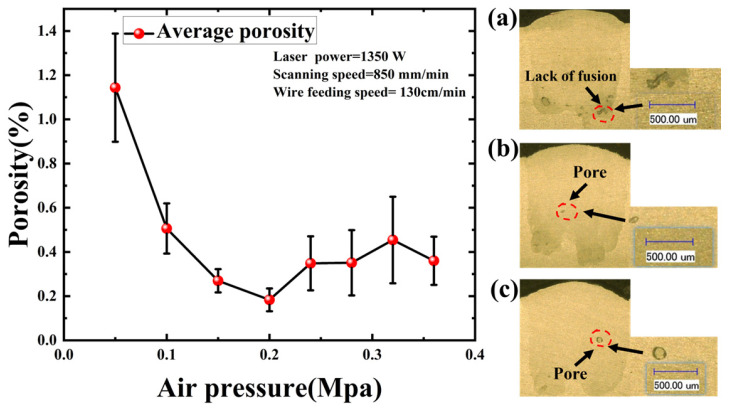
Influence of Air Pressure on Porosity. (**a**) 0.05 MPa; (**b**) 0.2 MPa; (**c**) 0.32 MPa.

As depicted in Figure 6, porosity levels were significantly elevated at 0.05 MPa. At this pressure, the shielding gas flux was insufficient to effectively suppress the plasma plume generated by the laser-material interaction, resulting in the accumulation of a dense plasma plume above the melt pool [39]. This plume attenuates laser energy via refraction and scattering, thereby reducing the effective energy density delivered to the substrate and wire. This energy deficit promotes the formation of lack-of-fusion defects. Concurrently, reduced melt pool fluidity exacerbates gas entrapment. Collectively, these factors led to increased porosity. As the air pressure increased, the effective suppression of the plasma plume enhanced laser energy transmission efficiency, resulting in decreased porosity. Minimum porosity was observed at 0.2 MPa. In this regime, shielding gas provided a dual benefit: maintaining an inert environment to preclude oxidation and generating a flow velocity sufficient to stabilize the plasma plume. However, pressures exceeding 0.2 MPa resulted in a renewed increase in porosity. Excessive air pressure exerts a significant mechanical force on the melt pool surface, inducing severe fluctuations and spatter. Furthermore, the high-velocity gas flow accelerates convective heat loss, thereby increasing the solidification rate [40]. These combined effects entrap gas within the melt pool before it can escape, leading to pore formation.

### 3.2. Orthogonal Experiment

Although single-factor experiments evaluated the independent effects of laser power, scanning speed, wire feeding speed, and air pressure on porosity, significant interaction effects exist in additive manufacturing processes. Consequently, relying solely on univariate analysis is insufficient to capture the complex coupling mechanisms governing porosity. To address this limitation, an orthogonal experimental design was employed to further optimize the process parameter combinations [41].

The parameters selected for the four-factor, four-level orthogonal experiment are detailed in Table 2. This experimental phase investigated the combined effects of laser power, scanning speed, wire feeding speed, and air pressure on porosity; the corresponding results are presented in Table 3. Analysis of the experimental data revealed that a minimum porosity of 0.13% was achieved under the following optimized conditions: a laser power of 1450 W, a scanning speed of 650 mm/min, a wire feeding speed of 130 cm/min, and an air pressure of 0.2 MPa.

Range analysis and variance analysis were used to comprehensively evaluate the influence of weight and the significance of process parameters on porosity. The range analysis results are shown in Table 4. The larger the range value (R), the more significant the effect of its numerical change on the experimental results. The relationship of range values (R) is: R _Laser Power_ > R _Wire Feeding Speed_ > R _Scanning Speed_ > R _Air Pressure_. Among them, laser power (R = 1.589) has the most significant effect on porosity, followed by wire feeding speed, while scanning speed and air pressure have relatively smaller effects on porosity. The variance analysis results are shown in Table 5, further confirming that laser power has a significant effect on porosity (F = 3.901 > F-critical = 3.490). This is mainly attributed to laser power as the core of energy input, which directly determines the stability of the keyhole bottom and the solidification rate of the molten pool. In terms of interaction effects, laser power and scanning speed (A × B) is a strongly significant interaction term (Sum of squares = 5.782, F = 16.05 > F-critical = 8.020). The two jointly determine molten pool stability and gas escape efficiency by regulating the balance between energy input and solidification rate. Laser power and wire feeding speed (A × C) is a marginally significant interaction term (F = 5.93, close to F-critical = 8.020). The interaction terms of scanning speed × wire feeding speed (B × C), laser power × air pressure (A × D), scanning speed × air pressure (B × D), and wire feeding speed × air pressure (C × D) are all insignificant (F values far less than 8.020), with weak effects on porosity. In summary, the range analysis and variance analysis results are highly consistent, clarifying that laser power and its interaction with scanning speed are key to controlling sample porosity. This provides a theoretical basis for process optimization.

## 4. Porosity Prediction Model

While single-factor experiments have elucidated the independent effects of process parameters and orthogonal experiments have identified optimal settings and significant interactions, limitations persist. The interplay of process parameters in additive manufacturing is inherently complex; relying on a finite set of discrete experimental data points is insufficient to fully capture the continuous, non-linear behavior of porosity. To address these challenges, this work integrates machine learning to establish a predictive model, utilizing a data-driven approach to quantify the complex mapping between process parameters and porosity [42].

This study employed four machine learning algorithms: Support Vector Regression (SVR), Random Forest (RF), Extreme Gradient Boosting (XGBoost), and Gaussian Process Regression (GPR). All models were implemented in a Python 3.9 environment using the scikit-learn library. The dataset containing 105 samples was randomly divided into training and testing sets at a ratio of 8:2, with a fixed random seed (random_state = 42) set to ensure reproducibility of results. Z-Score standardization [35] was applied to all input features to eliminate the effects caused by differences in magnitude between features. The formula is as follows.

(2) $$x^{\prime }=\frac{x-\mu }{\sigma }$$

where $x^{\prime }$ denotes the standardized feature value, x represents the original feature, and μ and σ correspond to the mean and standard deviation of the feature, respectively.

**Figure 7:**
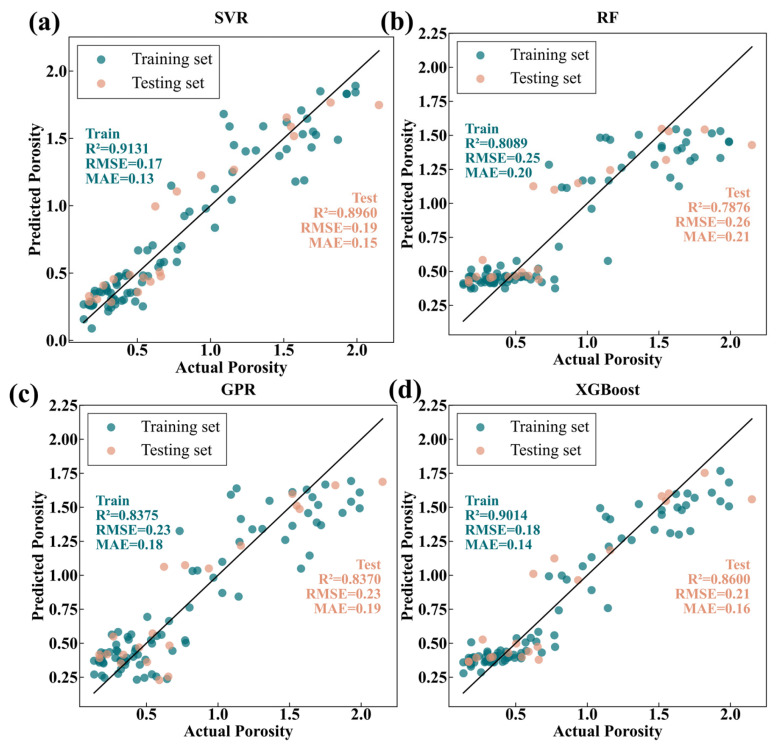
Comparison between Predicted and Observed Values for the Different Models. (**a**) SVR; (**b**) RF; (**c**) GPR; (**d**) XGBoost.

Hyperparameter optimization was performed on the training set using grid search combined with 5-fold cross-validation. The hyperparameter search space and optimal values for each model are shown in Table 6. Mean absolute error (MAE), root mean square error (RMSE), and coefficient of determination (R^2^) were selected as evaluation metrics for model performance. The calculation formulas are as follows.

(3) $$\mathrm{MAE}=\frac{1}{n}\sum_{i=1}^{n}\left|y_{i,\mathrm{measure}}-y_{i,\mathrm{predict}}\right|$$

(4) $$\mathrm{RMSE}=\sqrt{\sum_{i=1}^{n}\frac{{\left(y_{i,\mathrm{measure}}-y_{i,\mathrm{predict}}\right)}^{2}}{n}}$$

(5) $$R^{2}=1-\frac{\sum_{i=1}^{n}{\left(y_{i,\mathrm{measure}}-y_{i,\mathrm{predict}}\right)}^{2}}{\sum_{i=1}^{n}{\left(y_{i,\mathrm{measure}}-{\bar{y}}_{i}\right)}^{2}}$$

where n denotes the total sample size, while $y_{i,\mathrm{measure}}$ and $y_{i,\mathrm{predict}}$ represent the experimentally measured and predicted porosity values for the i specimen, respectively. The term ${\bar{y}}_{i}$ signifies the arithmetic mean of the observed porosity dataset. Figure 7 shows the comparison results between predicted porosity and actual porosity of the four machine learning models. The R^2^ difference between the training set and test set for all four models is less than 0.05, indicating that all models have no overfitting and possess good generalization ability. Among them, SVR (R^2^ = 0.8960, RMSE = 0.19, MAE = 0.15) shows the best overall performance, superior to the other three models. XGBoost (R^2^ = 0.8705, RMSE = 0.21, MAE = 0.15) and GPR (R^2^ = 0.8370, RMSE = 0.23, MAE = 0.19) perform second best. RF (R^2^ = 0.7876, RMSE = 0.26, MAE = 0.21) has the weakest prediction performance. The superiority of SVR is attributed to the RBF kernel function effectively capturing nonlinear relationships. The optimized regularization parameter (C = 10), kernel parameter (gamma = 0.1), and insensitivity parameter (epsilon = 0.1) achieve the best balance between model complexity and generalization ability.

**Figure 8:**
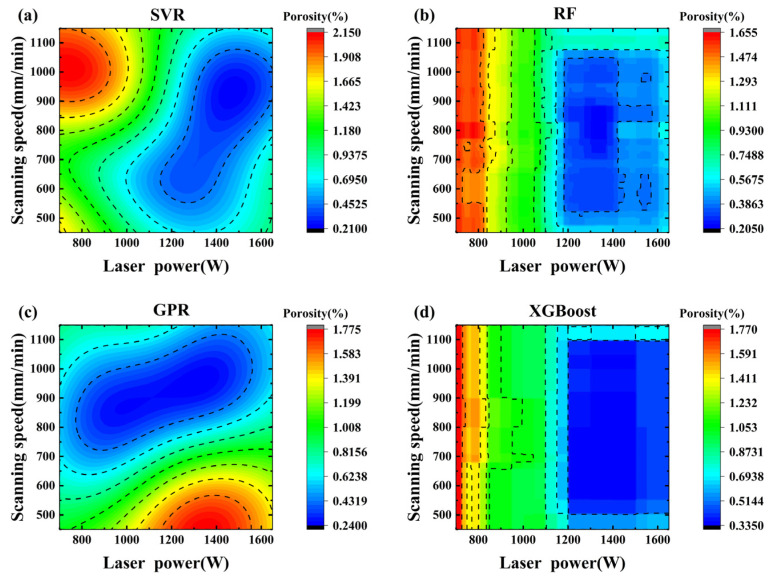
Porosity prediction maps generated by the different models at a wire feeding speed of 130 cm/min and an air pressure of 0.24 MPa. (The dashed black lines represent a set of contour lines corresponding to a constant value). (**a**) SVR; (**b**) RF; (**c**) GPR; (**d**) XGBoost.

To verify the prediction capability of machine learning models for the process window, Figure 8 shows the porosity prediction maps of the four machine learning models when laser power (700~1700 W) and scanning speed (450~1150 mm/min) vary under a fixed wire feeding speed of 130 cm/min and an air pressure of 0.24 MPa. The results show that the prediction results of the RF (Figure 8b) and XGBoost (Figure 8d) models have poor continuity [43], which can be attributed to their inherent characteristics as ensemble learning algorithms. The prediction results of SVR (Figure 8a) and GPR (Figure 8c) models have better continuity because they have stronger generalization capability than ensemble learning algorithms (such as RF and XGBoost) in handling small samples and nonlinear regression problems. Based on the above analysis, SVR demonstrates excellent performance in both performance metrics (R^2^ = 0.8960, RMSE = 0.19, MAE = 0.15) and prediction result continuity. Therefore, the SVR model is ultimately selected for constructing the porosity prediction map.

To further evaluate the effectiveness of the proposed SVR model, this study compared its performance with previous research using machine learning to predict porosity or relative density in additive manufacturing, as shown in Table 7. Compared with Ref (1) [44], which also focused on aluminum alloys, the present model demonstrates a significantly higher correlation (R^2^: 0.896 vs. 0.772). Additionally, although Ref (2) [26] achieved a similar R^2^ value in the wire arc additive manufacturing (WAAM) of steel, the current model has a lower mean square error (MSE: 0.036 vs. 2.06), demonstrating superior predictive stability.

Although some models for laser powder bed fusion (LPBF) of titanium alloys or stainless steel (Ref (3) [23] and Ref (4) [24]) obtained higher R^2^ values, the prediction accuracy achieved in this study (R^2^ = 0.896) remains significant considering the complexity of the LWDED process. Regarding material properties, aluminum alloys have higher reflectivity and thermal conductivity than titanium alloys or stainless steel, leading to instability in energy input to the molten pool, which increases the difficulty of porosity prediction. Regarding the process itself, unlike LPBF, LWDED involves complex laser-wire interactions where the molten metal transfer modes (such as bridge or globular transfer) act as inherent disturbance sources, resulting in a lower signal-to-noise ratio (SNR). Furthermore, the high solubility of hydrogen in high-temperature liquid aluminum promotes the formation of stochastic metallurgical pores, further introducing process noise. All these factors increase the difficulty of predicting porosity in the LWDED process.

**Figure 9:**
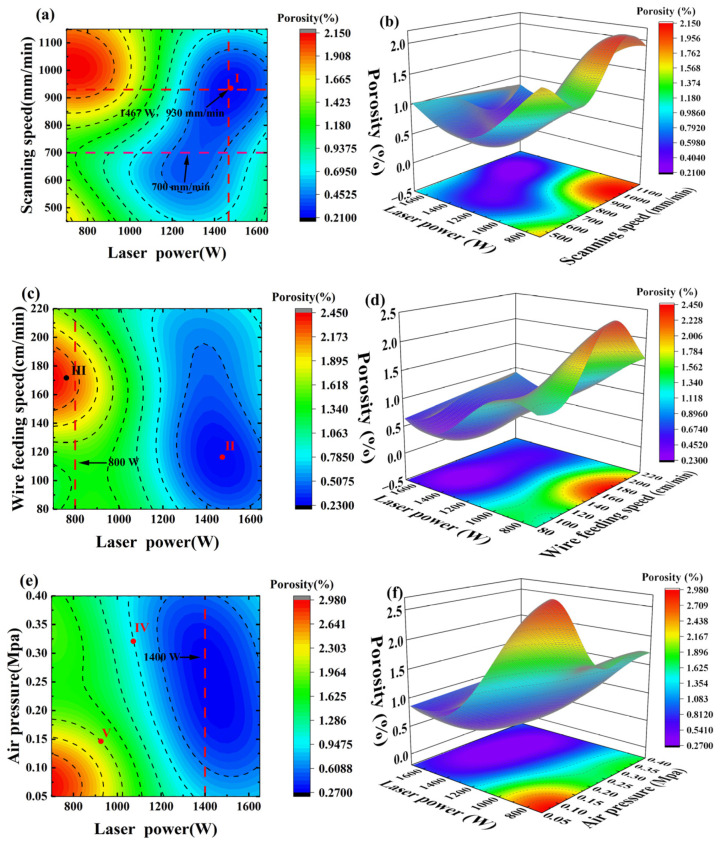
2D contour plots and 3D surface plots showing the interaction effects of process parameters on porosity. (**a**,**b**) interaction between laser power and scanning speed; (**c**,**d**) interaction between laser power and wire feeding speed; (**e**,**f**) interaction between laser power and air pressure.

Figure 9 presents the contour plots and 3D surface plots of the predicted porosity variation under multi-parameter interactions based on the SVR model. Figure 9a,b illustrates the interaction between laser power and scanning speed (Fixed wire feed speed at 130 cm/min and air pressure at 0.24 MPa). As shown in Figure 9a, the porosity reaches a minimum of 0.2186% when the laser power is 1467 W, and the scanning speed is 930 mm/min. This optimal combination falls within the range determined by the previous single-factor experiments (approximately 1350 W for laser power and 850 mm/min for scanning speed). The corresponding 3D surface plot in Figure 9b provides a more intuitive visualization of this trend, exhibiting a clear “valley” shape. Further analysis reveals that when the scanning speed is fixed at 700 mm/min, the porosity first decreases and then increases with increasing laser power, which is consistent with the influence pattern observed in the single-factor experiments. Figure 9c,d shows the interaction between laser power and wire feeding speed (Fixed scanning speed at 850 mm/min and air pressure at 0.24 MPa). As shown in Figure 9c, when the laser power is fixed at 800 W, the porosity increases with the wire feeding speed and reaches a peak at 170 cm/min, aligning with the single-factor results. Figure 9d further indicates that the combination of high wire feeding speed and low laser power leads to a significant increase in porosity, forming a prominent “peak” region on the response surface. Figure 9e,f demonstrates the interaction between laser power and air pressure (Fixed wire feed speed at 130 cm/min and scanning speed at 850 mm/min). As shown in Figure 9e, when the laser power is held constant at 1400 W, the porosity exhibits a U-shaped trend as air pressure increases, reaching its minimum at approximately 0.2 MPa. Figure 9f further shows that the combination of low laser power and low air pressure also results in a significant increase in porosity. In summary, the contour plots and 3D surface plots generated by the SVR model reveal the evolution of porosity under multi-parameter interactions. These results are consistent with the previous experimental conclusions, thereby verifying the reliability of the model.

To verify the effectiveness of the porosity prediction map constructed by the SVR model, five groups of typical process parameter combinations were selected from Figure 9 for validation experiments to compare measured values and predicted values. Table 8 shows the five groups of process parameter combinations. Table 9 shows the measured values, predicted values, and absolute errors (AE) corresponding to each group of parameters. As can be seen from Table 9, the maximum absolute error between measured values and true values does not exceed 0.514%, with an average absolute error of 0.251%, proving the effectiveness of the porosity prediction visualization map.

Although the contour plots constructed based on the SVR model intuitively reveal the interactions among process parameters, this method is constrained by the dimensionality reduction inherent in 2D visualization, requiring two parameters to be fixed. Consequently, the identified process window only represents a local optimum within a specific slice. To overcome this dimensional limitation and fully exploit the synergistic effects among multiple variables within a 4D continuous parameter space, this study establishes an SVR-GA optimization framework. The trained SVR model is defined as the objective function, aiming to minimize the predicted porosity. The constraints strictly follow the parameter ranges defined by the experimental design: laser power (700–1700 W), scanning speed (450–1150 mm/min), wire feed speed (80–220 cm/min), and air pressure (0.05–0.36 MPa). The core parameters of the Genetic Algorithm (GA) [45] are set as follows: population size = 30, maximum number of iterations = 1500, crossover probability = 0.7, and mutation probability = 0.2. The optimization results indicate that the system achieves a global optimum when the laser power is 1414.58 W, the wire feed speed is 113.99 cm/min, the scanning speed is 831.76 mm/min, and the air pressure is 0.18 MPa. Under this optimal parameter combination, the minimum porosity predicted by the model is further reduced to 0.127%. This marks the transition from visual analysis to automated algorithmic optimization.

Although the dataset in this study originates from experimental characterization of the 5356-aluminum alloy, the proposed modeling framework exhibits significant transferability and generalizability. The predictive reliability of this framework is grounded in the steady-state physical mechanisms inherent to the LWDED process: as long as a stable molten pool and molten metal transfer modes are maintained during deposition, and the input parameters remain within the calibrated valid range of the model, this data-driven approach can deliver reliable predictions. Therefore, this methodology offers a universal reference value for process optimization in other metallic systems, such as titanium alloys and stainless steels.

## 5. Conclusions

In LWDED technology, low-porosity 5356 aluminum alloy samples were prepared by optimizing process parameters. Experiments clarified the influence patterns of process parameters on porosity and the significance of interaction terms. Based on machine learning models, contour maps and 3D surface plots were established to study the effects of process parameters on porosity in depth. The conclusions are summarized as follows:

(1) Single-factor experiments were conducted to clarify the independent influence patterns of four process parameters—laser power, scanning speed, wire feeding speed, and air pressure—on porosity. Through orthogonal experiments combined with range analysis and variance analysis, the influence degree of each process parameter on porosity was determined in descending order: laser power > wire feeding speed > scanning speed > air pressure. Multi-parameter interactions are equally important in controlling porosity. Among them, laser power × scanning speed is a strongly significant interaction term (F = 16.05 > 8.020).
(2) Porosity prediction models were constructed using four machine learning algorithms: SVR, RF, GPR, and XGBoost. Comprehensive comparison showed that the SVR model performed best in porosity prediction (R^2^ = 0.8960, RMSE = 0.19, MAE = 0.15).
(3) Focusing on the interactions between laser power and scanning speed, wire feeding speed, and air pressure, contour maps and 3D surface plots based on the SVR model were constructed. These present the variation patterns of porosity under multi-parameter interactions, and the porosity variation trends are consistent with experimental conclusions. Validation experiments showed that the maximum prediction error does not exceed 0.514%, with an average error of 0.251%, demonstrating good model reliability.

In summary, this study intuitively reveals the variation law of porosity under the multi-parameter interaction during the LWDED process through experiments, machine learning modeling, and visualization, providing a visual tool for the rapid selection of low-porosity process parameter combinations for the LW-DED technology.

## Figures and Tables

**Table 1:** Chemical composition of the 5356 aluminum alloy (wt.%) [30].

Element	Al	Mg	Mn	Cr	Cu	Zn
Al5356	≥93	4.5~5.5	0.05~0.20	0.05~0.20	≤0.10	≤0.10

**Table 2:** Experimental Parameters and Levels.

Parameters	Range
Laser power (W)	700, 950, 1200, 1450
Scanning speed (mm/min)	450, 650, 850, 1050
Wire feeding speed (cm/min)	80, 110, 140, 170
Air pressure (MPa)	0.05, 0.2, 0.32, 0.4

**Table 3:** Orthogonal Experimental Design Layout and Corresponding Porosity Results.

No	LP (W)	SS (mm/min)	WFS (cm/min)	AP (MPa)	Average (%)
1	700	450	80	0.05	1.99
2	700	650	110	0.2	1.52
3	700	850	140	0.32	1.75
4	700	1050	170	0.4	1.80
5	950	450	80	0.05	0.86
6	950	650	110	0.2	0.62
7	950	850	140	0.32	0.77
8	950	1050	170	0.4	0.82
9	1200	450	80	0.05	0.30
10	1200	650	110	0.2	0.26
11	1200	850	140	0.32	0.37
12	1200	1050	170	0.4	0.39
13	1450	450	80	0.05	0.19
14	1450	650	110	0.2	0.13
15	1450	850	140	0.32	0.21
16	1450	1050	170	0.4	0.30

Note: LP: laser power, SS: scanning speed, WFS: wire feeding speed, AP: air pressure.

**Table 4:** Results of Range Analysis for the Orthogonal Experiment.

Parameter	Level 1	Level 2	Level 3	Level 4	Range (R)
Laser power (W)	1.797	0.768	0.331	0.208	1.589
Scanning speed (cm/min)	0.821	0.744	0.752	0.787	0.077
Wire feeding speed (mm/min)	0.834	0.634	0.774	0.862	0.228
Air pressure (MPa)	0.822	0.725	0.791	0.765	0.097

**Table 5:** Results of Analysis of Variance (ANOVA) for the Orthogonal Experiment.

Source of Mutation	Sum of Squares	Degree of Freedom	F Value	F Critical Value
Main effect				
Laser power (A)	6.256	3	3.901	3.490
Scanning speed (B)	0.015	3	0.009	3.490
Wire feeding speed (C)	0.124	3	0.077	3.490
Air pressure (D)	0.020	3	0.012	3.490
Interaction effect				
Laser power × Scanning speed (A × B)	5.782	9	16.05	8.020
Laser power × Wire feeding speed (A × C)	2.136	9	5.93	8.020
Laser power × Air pressure (A × D)	0.152	9	0.43	8.020
Scanning speed × Wire feeding speed (B × C)	0.218	9	0.60	8.020
Scanning speed × Air pressure (B × D)	0.089	9	0.22	8.020
Wire feeding speed × Air pressure (C × D)	0.067	9	0.17	8.020

**Table 6:** Hyperparameter tuning of machine learning models.

Classifiers	Hyperparameters	Optimal Values	Range Studied
SVR	kernel	‘rbf’	[‘rbf’]
	regularization parameter (C)	‘10’	[0.1, 1, 10]
	gamma	‘0.1’	[‘scale’, 0.01, 0.1]
	epsilon	‘0.1’	[0.1, 0.2, 0.5]
RF	n_estimators	‘120’	[80, 100, 120]
	max_depth	‘4’	[4, 5, 6]
	min_samples_split	‘10’	[10, 15, 20]
	min_samples_leaf	‘5’	[5, 7, 10]
	max_features	‘sqrt’	[‘sqrt’]
GPR	alpha	‘0.1’	[0.3, 0.5, 0.8, 1.0]
	n_restarts_optimizer	‘10’	[‘10’]
	kernel	‘rbf’	[‘rbf’]
XGBoost	learning_rate	‘0.03’	[0.01, 0.02, 0.03]
	Max_depth	‘3’	[3, 4]
	n_estimators	‘120’	[80, 100, 120]
	subsample	‘0.6’	[0.6, 0.7, 0.8]
	colsample_bytree	‘0.8’	[0.6, 0.7, 0.8]

**Table 7:** Performance comparison between the proposed model and existing literature models.

Study	Process	Materials	ML Model	MSE	R^2^
Ref (1) [44]	LPBF	AlSi10Mg	ANN	0.232	0.772
Ref (2) [26]	WAAM	ER70S6	SVR	2.06	0.898
Ref (3) [23]	LPBF	Ti6Al4V	ANN	5.981 × 10^−6^	0.97
Ref (4) [24]	LPBF	martensitic SS	GBDT	0.158	0.983
This Work	LWDED	Al5356	SVR	0.036	0.896

**Table 8:** Process Parameters Selected for the Validation Experiments.

Experiment	Laser Power (W)	Scanning Speed (mm/min)	Wire Feeding Speed (cm/min)	Air Pressure (MPa)
I	1480	938	130	0.24
II	1470	850	116	0.24
III	750	900	170	0.24
IV	1050	850	130	0.32
V	920	850	130	0.15

**Table 9:** Measured vs. Predicted Values using the SVR Model.

Experiment	Measured Value (%)	Predicted Value (%)	AE
	SVR		
I	0.3245	0.2219	0.1026
II	0.2891	0.2415	0.0476
III	2.8346	2.3209	0.5137
IV	1.2314	0.9475	0.2839
V	1.6541	1.9616	0.3075

## Data Availability

The original contributions presented in this study are included in the article. Further inquiries can be directed to the corresponding author.
